# Death of M. Dubois

**Published:** 1837-07-01

**Authors:** 


### 5. Death of M. Dubois.

This pupil of Dessault, and friend of Bichat and Lavoisier, died at Paris, on the 30th of March, in the eighty-first year of his age.

